# Impact of childhood trauma and affective temperament on resilience in bipolar disorder

**DOI:** 10.1186/s40345-015-0023-3

**Published:** 2015-02-24

**Authors:** Sermin Kesebir, Başak Ünübol, Elif Tatlıdil Yaylacı, Duru Gündoğar, Hüseyin Ünübol

**Affiliations:** Psychology Department, Faculty of Humanities and Social Sciences, Üsküdar University, Altunizade Mh., Haluk Türksoy Sk. No. 14, Üsküdar, Istanbul, 34662 Turkey; Erenköy Psychiatry Education and Research Hospital, Kadıköy, Istanbul, 34216 Turkey; Psychiatry Department, Ankara Numune Education and Research Hospital, Altındağ, Ankara, 06100 Turkey; Psychiatry Department, School of Medicine, Süleyman Demirel University, Çünür Mh., Süleyman Demirel Caddesi, Isparta, 32260 Turkey; Institute of Health Sciences, Üsküdar University, İcadiye Mh., Bağlarbaşı Cd. No. 63, Üsküdar, Istanbul, 34674 Turkey

**Keywords:** Bipolar disorder, Childhood trauma, Affective temperament, Resilience

## Abstract

**Background:**

The aim of this study was to investigate whether childhood trauma (CT) and affective temperament have an impact on resilience in bipolar patients.

**Methods:**

One hundred cases with bipolar disorder (BD) diagnosis according to the Diagnostic and Statistical Manual of Mental Disorders, 4th Edition (DSM-IV) were evaluated consecutively in their euthymic period during outpatient follow-up interviews. Diagnostic interviews were done with SCID-I, affective temperament was evaluated with the Temperament Evaluation of Memphis, Pisa, Paris and San Diego Autoquestionnaire, and resilience was evaluated with the Resilience Scale for Adults (RSA). The presence of CT was determined and measured with the Childhood Trauma Questionnaire (CTQ).

**Results:**

Among the bipolar patients, it was found that 35 cases (35%) were CT+. Depressive, cyclothymic, and anxious temperament scores were higher in CT+ cases. However, resilience scores were higher in CT− cases. In bipolar patients with and without childhood trauma, the relationship between temperament and resilience appears to be different. A negative relation between sexual abuse, emotional abuse, emotional neglect, and anxious temperament scores and resilience scores was shown in regression analysis.

**Conclusions:**

CT and affective temperament both have an impact on resilience in bipolar patients.

**Electronic supplementary material:**

The online version of this article (doi:10.1186/s40345-015-0023-3) contains supplementary material, which is available to authorized users.

## Background

In the 1970s, researchers noticed that some individuals could pass through normal developmental processes despite adverse circumstances and they started to investigate the neurobiological determinants as well as the psychosocial determinants in these individuals (Feder et al. 2009; Fadardi et al. 2010; Amico et al. 2011). Psychological resilience can be defined as the capability to adapt in adverse environmental circumstances (Basım and Çetin 2011) and is determined by individual characteristics, family cohesion and support, and external support systems (Rutter 1985). In the scale developed to evaluate psychological resilience (Friborg et al. 2003), personal strength, structural style, social competence, family cohesion, and social resources subdimensions are investigated. Personal strength subdimension is further divided into perception of self and perception of future (Friborg et al. 2005). In Simeon et al.’s study in 2007, in healthy individuals, childhood trauma was identified as the leading determinant of psychological resilience, and there was a strong inverse relationship between the two.

The affective temperament of an individual is one of the structural, biological, and genetic factors determining the risk of depression and mania (Angst 2000). It was claimed that there was a continuity between affective temperament and mood disorders, and it was demonstrated in numerous studies that there was a relationship between affective temperament and the clinical course of mood disorders (Kesebir et al. 2005; Rihmer et al. 2010). Among the environmental factors affecting the etiology of mood disorders, childhood trauma (CT) is one of the leading factors (Simeon et al. 2007). Similar to clinical course, age of onset, and episode severity, CT is a factor that affects the cross-sectional phenomenology of mood disorders.

In one of our previous studies, we demonstrated that resilience was related to affective temperament in major depressive disorder (MDD) cases (Kesebir et al. 2013). We also found that this relationship was different between CT+ and CT− patients (Gündoğar et al. 2014). In this study, we aimed to investigate if childhood trauma and affective temperament have an impact on resilience in bipolar patients.

## Methods

### Sample and procedure

In this study, a total of 100 patients (54 females and 46 males) aged between 17 and 77 (mean age 32.7 ± 13.2), who were admitted to the outpatient clinic of Erenköy Psychiatry Education and Research Hospital for their regular follow-up interviews, have been followed regularly for at least 1 year, and were diagnosed with bipolar disorder (BD) type 1 according to the Diagnostic and Statistical Manual of Mental Disorders, 4th Edition (DSM-IV), were recruited consecutively. Recruitment of patients was done between August 2013 and August 2014. Approval for the study was obtained from Erenköy Psychiatry Hospital Training and Scientific Investigations Committee in compliance with the Helsinki Declaration. All the patients were in remission and gave written informed consent to attend the study. Our sample consisted of middle-class subjects who live in the city center. As a limitation, our study lacks a control group.

Diagnostic interviews were performed with the Turkish version of SCID-I (Çorapçıoğlu et al. 1999). Cases with comorbid psychiatric diagnoses were excluded from the study. Comorbidity, similar to severity of disease, was evaluated as a variable which is predicted by childhood trauma and decreased resilience. The requirement for being in remission was identified as obtaining a score of less than 8 from the Hamilton Depression Rating Scale (HDRS). The mean duration of remission at the time of evaluation of the cases was 8.6 ± 3.1 weeks.

After this initial evaluation, the Turkish version of the Temperament Evaluation of Memphis, Pisa, Paris and San Diego Autoquestionnaire (TEMPS-A) and the Resilience Scale for Adults (RSA)-Turkish version were administered. The presence of CT was determined and measured with the Childhood Trauma Questionnaire (CTQ). Invalid forms are excluded.

### Assessment tools

The RSA was developed by Friborg et al. (2003). A higher total score obtained from the items scored between 1 and 5 indicates that the psychological resilience of the individual is higher. Turkish reliability and validity study was carried out by Basım and Çetin (2011).

The TEMPS-A Temperament Scale was developed by Akiskal for evaluating affective temperament (Akiskal et al. 2005). The questionnaire consists of 100 items determining the depressive, hyperthymic, irritable, cyclothymic, and anxious temperaments. The reliability and validity study in Turkish version was done by Vahip et al. (2005).

CTQ was developed by Bernstein et al. (1997) to evaluate the existence and type of childhood traumas. Its reliability and validity study in Turkish was carried out by Şar et al. (2012).

### Statistical analysis

Statistical analysis was performed with SPSS version 20.0. Categorical variables were compared by the chi-square test while numerical variables were compared by the Mann-Whitney *U* test. The Pearson correlation test was used for correlation analysis. Linear regression analysis was applied to strengthen the correlation analysis. Statistical significance was set at <0.05 and all tests were two-tailed.

## Results

The mean age of 54 female and 46 male patients was 32.7 ± 13.2. All of the patients were bipolar disorder type 1. The frequency of illness (Number of total episode/Duration of illness − Year) was 1.9 ± 1.1.

Among the bipolar patients, it was found that 35 cases (35%) (22 females, 13 males) were CT+.

### Comparison of affective temperament and resilience in CT+ vs CT−

Depressive, cyclothymic, and anxious temperament scores were higher in CT+ cases. However, resilience scores were higher in CT− cases (Table 1).

**Table 1:** **Comparison of affective temperament and resilience in CT+ vs CT− patients**

	**CT+**	**CT−**	**Analysis (** ***p*** **)**
Resilience	123.7 ± 23.3	140.2 ± 19.7	*0.004*
Depressive temperament	9.8 ± 4.6	5.7 ± 4.2	*0.001*
Cyclothymic temperament	10.1 ± 4.9	6.9 ± 5.5	*0.020*
Hyperthymic temperament	9.3 ± 5.6	11.5 ± 5.8	0.112
Irritable temperament	5.4 ± 4.0	4.1 ± 3.7	0.190
Anxious temperament	8.1 ± 5.8	4.2 ± 4.4	*0.003*

Significant results are shown in italic font.

### The relationship between affective temperament and resilience

There was a weak inverse relationship between resilience scores and anxious temperament scores in CT+ bipolar patients (Table 2).

**Table 2:** **The relationship between affective temperament and resilience**

	**CT+ ** **(** ***r*** **,** ***p*** **)**	**CT− ** **(** ***r*** **,** ***p*** **)**
Depressive temperament	−0.062, 0.696	−0.274, 0.146
Cyclothymic temperament	−0.188, 0.227	−0.230, 0.152
Hyperthymic temperament	0.173, 0.268	0.285, 0.140
Irritable temperament	−0.197, 0.206	−0.313, 0.192
Anxious temperament	−*0.279*, *0.050*	−0.210, 0.293

Significant results are shown in italic font.

### The relationship between CT and resilience

There was a moderate inverse correlation between emotional abuse, sexual abuse, emotional neglect, and resilience in CT+ cases (Table 3).

**Table 3:** **The relationship between CT and resilience**

	**(** ***r*** **,** ***p*** **)**
Physical neglect	−0.221, 0.154
Emotional neglect	−*0.337*, *0.050*
Emotional abuse	−*0.502*, *0.040*
Physical abuse	−0.182, 0.242
Sexual abuse	−*0.423*, *0.042*

Significant results are shown in italic font.

### The relationship between CT and affective temperament

There was a weak correlation between physical and emotional neglect and anxious temperament in CT+ cases (Table 4). There was a weak inverse correlation between emotional abuse and neglect and hyperthymic temperament.

**Table 4:** **The relationship between CT and affective temperament**

	**Depressive temperament ** **(** ***r*** **,** ***p*** **)**	**Cyclothymic temperament ** **(** ***r*** **,** ***p*** **)**	**Hyperthymic temperament ** **(** ***r*** **,** ***p*** **)**	**Irritable temperament ** **(** ***r*** **,** ***p*** **)**	**Anxious temperament ** **(** ***r*** **,** ***p*** **)**
Physical neglect	0.318, 0.138	0.235, 0.229	−0.162, 0.298	0.007, 0.965	*0.371*, *0.026*
Emotional neglect	0.347, 0.123	0.220, 0.257	−*0.305*, *0.049*	−0.062, 0.695	*0.292*, *0.034*
Emotional abuse	0.314, 0.140	0.118, 0.451	−*0.348*, *0.045*	−0.021, 0.893	0.071, 0.652
Physical abuse	0.308, 0.145	0.167, 0.283	−0.158, 0.311	0.081, 0.604	0.144, 0.356
Sexual abuse	0.269, 0.151	0.181, 0.246	−0.098, 0.532	−0.056, 0.720	−0.025, 0.873

Significant results are shown in italic font.

### Regression analysis

A negative relation between sexual abuse, emotional abuse, emotional neglect, and anxious temperament scores and resilience scores was shown in regression analysis (respectively, beta = −5.412, *p* = 0.010; beta = −3.918, *p* = 0.025; beta = −3.238, *p* = 0.054 vs beta = −3.715, *p* = 0.039). The variables explained 72% of the total variance (*R*^2^ = 144.87, *n* = 100).

## Discussion

As far as we know, this is the first study that investigates the relationship between resilience, CT, and temperament in bipolar patients. Our results suggest that CT+ patients are less resilient than CT− patients. The inverse relationship between resilience scores and CT scores was found significant for emotional neglect, emotional abuse, and sexual abuse. This relationship was more prominent for emotional and sexual abuse. The negative effect of all three CT types on resilience was further affirmed by the results of the regression analysis. Interestingly, no relation was detected between physical abuse, physical neglect, and resilience scores. However, previously, physical abuse had been reported as a predictive risk factor for mania (Levitan et al. 1998).

On the other hand, the findings provide a great deal of support for an additive or main effect perspective on vulnerability and protective factors and some support for an interactive perspective. It appears that some protective and vulnerability factors do not have stronger effects for physically abused children, but instead are equally beneficial or harmful to children regardless of their abuse status (Lansford et al. 2006). Childhood physical abuse is often associated with detrimental physical and psychological consequences in adulthood. Some adults appear to overcome effects of very severe parental physical abuse in childhood. Pitzer and Fingerman (2010) considered whether psychosocial resources explain variability in well-being for adults who experienced childhood physical abuse by their parents. High levels of personal control were associated with better physical and psychological functioning among adults who were physically abused as children. Thus, personal control may be a key factor to health and well-being and thus resilient functioning following childhood abuse.

According to our results, affective temperament scores also differ between subjects with or without CT. Depressive, cyclothymic, and anxious temperament scores were higher in bipolar patients with CT. However, only anxious temperament scores were found to be associated with resilience. This association is an inverse one. To add to the weight of our results, there was a linear relation between CT and affective temperament, anxious temperament, physical neglect, and emotional neglect scores.

The relationship between temperament and CT is most often reported between anxious temperament and CT (Ogawa et al. 1997). Biological markers were also suggested for this relationship (Alm and Risberg 2007). In the presence of CT, it was shown that anxious temperament increases the risk for a mood disorder, and this risk was claimed to be associated with CRH receptor gene polymorphism (Rogers et al. 2013). Negative emotionality being the common denominator of the abovementioned three affective temperament types, our results indicate that negative emotionality becomes more prominent in the presence of childhood trauma.

There was an inverse relation between psychological resilience and harm avoidance (HA) in Simeon et al.’s (2007) study, and HA avoidance was determined to be one of the negative predictors of psychological resilience. HA is a temperament quality related to anxious temperament. Passive avoidance which is a very prominent quality of anxious temperament (Degnan and Fox 2007) has a negative effect on resilience (Clark 2005). An internal locus of control is an important variable which increases resilience (Werner 1992). Individuals with anxious temperaments are dependent on an external locus of control (Rutter 2002). Prompt and intense emotionality observed in this situation is a risk factor especially in subclinical and chronic depressions (Purper-Ouakil et al. 2002).

In our previous study, hyperthymic temperament scores which were lower than those of healthy individuals did have a strong relationship with psychological resilience scores which were also lower than those of healthy individuals (Kesebir et al. 2013). Based on the strength of this relationship, we suggested that protective factors may play a more important role than risk factors in resilience. In this study, we found an inverse relation between hyperthymic temperament scores and emotional abuse and emotional neglect scores. We think that this relationship is supported by reward dependence (RD). Hence, RD is a variable which has a linear relation with psychological resilience (Simeon et al. 2007). However, in a study conducted with heroine-dependent individuals, physical neglect and emotional abuse were found to be associated with low RD (Evren et al. 2012).

Lack of a comparative control group is a limitation of this study. It should have been asked whether childhood trauma and affective temperament had an effect on resilience in healthy subjects as well. In addition, there are methodological difficulties in measuring temperament and resilience. It is not easy to differentiate the state and trait qualities between affective temperament and mood disorders. In this study, even though the patients were evaluated as ‘in remission’ according to DSM-IV, it is difficult to distinguish this ‘relative’ remission period from subsyndromal and subthreshold forms. Residual and subthreshold symptoms of bipolar disorder are usually related to the depressive pole (Samalin et al. 2014). Therefore, we intentionally administered only HDRS. Another limitation of this study is that the measurements for temperament and resilience and the determination of CT were based on the patients’ declaration. Validity would be higher if the semi-structured version of these assessment tools is available. Additionally, future studies could be designed as to include biological indicators of the mentioned concepts.

## Conclusions

In conclusion, personal characteristics which can increase liability for some pathologies may also be preventive for certain adverse events and increase resilience. The preventive or predisposing quality of a certain personal characteristic is determined by numerous other variables besides the nature of the experienced event. In other words, this effect is not invariable and constant. CT and affective temperament have an impact on resilience in bipolar patients. In individuals at risk, resilience itself can be a treatment goal. It can be intervened and is a relatively easy target.
